# Why Should Return to Sport Be Delayed by up to Two Years After ACL Reconstruction? A Narrative Review of the Biological, Surgical and Rehabilitation Evidence

**DOI:** 10.3390/jcm14165699

**Published:** 2025-08-12

**Authors:** Sebastiano Vasta, Pierangelo Za, Giuseppe Massazza, Ugo Riba, Alessandro Scotto di Palumbo, Kristian Samuelsson, Alexandra Horvath, Arrigo Giombini

**Affiliations:** 1 Fondazione Policlinico Universitario Campus Bio-Medico, 00128 Roma, Italy; 2 Research Unit of Orthopedics and Trauma Surgery, Department of Medicine and Surgery, Università Campus Bio-Medico di Roma, 00128 Roma, Italy; 3 Department of Movement, Human and Health Sciences, Foro Italico University of Rome, 00135 Rome, Italy; 4 Department of Surgical Sciences, University of Turin, 10126 Turin, Italy; 5 IRR Rehabilitation Center, 10126 Turin, Italy; 6 Department of Orthopaedics, Institute of Clinical Sciences, Sahlgrenska Academy, University of Gothenburg, 40530 Gothenburg, Sweden; 7 Department of Orthopaedics, Sahlgrenska University Hospital, 41345 Mölndal, Sweden; 8 Sahlgrenska Sports Medicine Center, 40530 Gothenburg, Sweden

**Keywords:** anterior cruciate ligament reconstruction, acl injury, proprioception, neuromuscular training, return to play

## Abstract

**Background:** Despite outstanding clinical outcomes are routinely achieved after ACL reconstruction (ACLR), the major current issue is the failure rate (re-rupture or objective clinical instability). Reinjury rates have been reported to be about 6% for ipsilateral graft rupture and 8% for contralateral ACL rupture, with a cumulative reinjury rate of about 20%. **Methods:** A comprehensive review of the literature was performed to summarize the latest evidence on biological, surgical and rehabilitation aspects of ACLR. **Results:** It has been demonstrated that young age is a risk factor for ACL graft rupture and so is not passing return-to-play (RTP) testing following ACLR (those who pass the RTP test battery have a one-third reduction in the ACL re-rupture rate). Furthermore, up to 30% of reinjury occurs within two years from ACLR. These data can be explained by numerous pieces of evidence showing that the recovery of proprioception, proper neuromuscular activation and strength, as well as proper biomechanics, remains affected for a long time after surgery (up to two or three years in some cases) despite adequate rehabilitation programs. **Conclusions:** Clinical evidence, together with biological data on the ligamentization process and the remodeling phase, suggest that return to strenuous sports, especially in younger athletes, should be delayed by at least 18 months or 2 years after ACLR.

## 1. Introduction

Anterior cruciate ligament (ACL) rupture is a common complication of distractive knee injuries, causing a high number of anterior cruciate ligament reconstruction (ACLR) surgeries. ACLR is the first sports medicine procedure performed in the United States [1] and has shown a tendency to increase over the years [2,3]. Despite a downward trend in new cases of ACL ruptures, the rate of ACL reconstruction has grown. This may reflect increased functional demands from patients and potential changes in surgical indications [4]. The incidence of ACL injuries seems to vary between studies, with some reporting 30 injuries per 100,000 individuals [3] and others reporting as many as 78 per 100,000 [5,6,7,8,9]. About a quarter of knee injuries occur during sports activities [10], and about a third of all sports injuries involve the knee [11]. Interestingly, the majority of ACL ruptures are sustained during non-contact activities [12]. The target population is predominantly male and under the age of 30 years [3]. While males tend to participate in sports activities to a higher extent, females have a two-to-eight times higher risk than their male counterparts to sustain an ACL rupture [13]. It has been hypothesized that certain anatomical features, including increased joint laxity, knee valgus and recurvatum, as well as hormonal effects on the ACL, put the female sex at higher risk of rupture [14,15]. Injury to the ACL is also reported in the pediatric population, with a prevalence ranging from 0.5% to 3% of ACL tears [16,17], leading to severe long-term sequelae on the menisci and cartilage [18,19,20]. Unfortunately, isolated ACL injuries are uncommon, with a high proportion of associated injuries reported, including chondral and meniscal injuries and involvement of other knee ligaments [21,22,23,24]. Despite good clinical outcomes after ACLR, the major current issue is the failure rate (re-rupture or objective clinical instability), which can reach up to 30% [25]. Achieving the objective of lowering this rate necessitates a thorough examination of risk factors, selecting an appropriate surgical approach, addressing associated injuries and implementing a tailored rehabilitation program.

Reinjury rates have been reported to be about 6% for ipsilateral graft rupture and 8% for contralateral ACL rupture [26], with a cumulative reinjury rate of about 20% [26,27]. It has been demonstrated that young age is a risk factor for ACL graft rupture [28] and so is not passing return-to-play (RTP) testing following ACLR [29]. In addition, those who fail to pass the RTP test and suffer a graft re-rupture do so at a much earlier time following surgery than those who pass their first RTP test [29]. Therefore, it seems that time from surgery to return to sports activity plays a role in determining the re-rupture rate. Previous research has shown that ACL graft tear (re-rupture) occurs within the first two years from surgery [30,31].

Psychological factors such as fear of reinjury, low self-confidence and poor motivation have a significant impact on RTS after ACLR [29,30]. Evidence suggests that athletes who score high on psychological readiness scales—such as the ACL-Return to Sport after Injury (ACL-RSI) scale—are more likely to achieve high scores in physical RTS tests and to resume sport at their preinjury level. Conversely, athletes with low psychological test scores tend to have a reduced likelihood of returning to sport, even when physical recovery appears adequate [32].

Among the most common psychological barriers are kinesiophobia and fear of reinjury, often present despite the absence of objective structural abnormalities. These psychological components may pose a greater obstacle than functional deficits themselves, highlighting the importance of including psychological support as part of a multidisciplinary rehabilitation approach [33].

Clinical evidence, together with biological data on the ligamentization process and the remodeling phase [34], suggests that return to strenuous sports, especially in younger athletes, should be delayed by at least 18 months or 2 years after ACLR. This review aims to summarize the latest evidence on biological, surgical and rehabilitation aspects of ACLR supporting slower RTP in favor of longer, more rigorous neuromuscular training.

## 2. Biology

**Figure 1:**
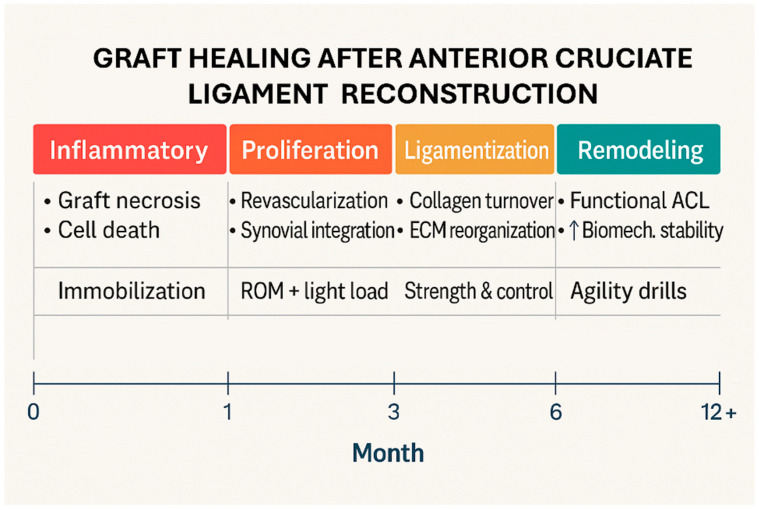
Visual timeline of graft healing. ↑ = increased.

Graft remodeling is the process through which the graft undergoes changes in its biological and mechanical properties until it partially acquires those of a native ACL. Typically, three stages of graft remodeling after ACL reconstruction are described [35], with the first phase involving an early graft healing phase characterized by necrosis and poor vascularization, lasting for four weeks. During the following eight weeks, proliferation occurs, during which the graft becomes revascularized. The final phase is the “*ligamentization*”, a term indicating biological and mechanical changes in the graft to acquire the characteristics of a ligament in order to replicate the properties of a native ACL. The process of *ligamentization* lasts up to 12 months, at which point the mechanical properties of the neoligament reach their peak [6,36], (Figure 1).

Two previous studies on ACLR report that the graft showed to be “very similar” to a normal ACL under light microscopy 24 months after surgery, while no further changes were evident after that time point [37,38]. Since biological failure has been described as a possible cause of retear [39], adhering to the timelines of biology is key to reducing the failure rate. This timeline prompts some considerations: Firstly, abiding by biology entails waiting for 12 months before having a neoligament similar to a native ACL. Speculatively, the knee should not be stressed before this time. However, we routinely witness aggressive rehabilitation protocols with return to sports activity as early as six to nine months after surgery depending on the type of sport. Secondly, early healing can be achieved by modifying the remodeling process just described. Some authors speculate that preserving the insertion of the hamstring tendon (HT) may bypass the necrosis phase [40,41,42]. Indeed, preserving the insertion of the HT allows for the early vascularization of the graft by the medial inferior geniculate artery, improving the graft’s remodeling process [43,44]. As mentioned earlier, when detaching the HT from the distal insertion, the necrosis phase lasts for 4 weeks, followed by 8 weeks of revascularization, totaling 12 weeks. It follows that during these initial 12 weeks, the graft is particularly vulnerable and therefore at high risk of rupture. Furthermore, the negative effects of necrosis may be persisting even after this period, undermining the mechanical properties of the graft [36,44,45]. Preserving the HT distal insertion and bypassing the necrosis phase would potentially mean gaining 12 weeks biologically and overcoming a period of particular weakness of the graft, with beneficial effects on mechanical strength and graft survival. Since performing a biopsy on the neoligament is ethically unsound, magnetic resonance imaging (MRI) has been widely utilized to study the graft healing process [46,47]. Signals from MRI show both graft integration within the bone tunnel and maturation of the intra-articular portion of the graft [47,48,49]. Howell et al. [50,51] were among the pioneers in suggesting an MRI grading system for assessing graft maturity in humans. Changes in signal intensity on MRI represent variations in the mechanical properties of the graft, load to failure and tensile strength and are therefore associated with the progress of the remodeling and incorporation process of the graft [52,53,54]. These changes continue for up to one year after ACLR and are particularly evident between six and nine months after surgery [55]. Vari et al. [56] analyzed the factors influencing ACL graft remodeling using the signal-to-noise quotient (SNQ) measured on MRI, demonstrating that leaving the HT distal insertion attached was significantly associated with better graft remodeling at a 1-year follow-up [56]. There were no significant correlations observed between the SNQ value and sex, body mass index, smoking habits or type of sports activity practiced postoperatively [56]. The authors attributed the improved outcomes to enhanced vascularization and dual fixation at the tibia [41,57], which enhances the pullout strength of the graft [42,58]. Grassi et al. [40] first demonstrated that better outcomes on MRI are observed both in the intra-articular portion and within the tibial tunnel portion of the graft when the insertion of the HTs is preserved as early as 4 months after surgery and up to 18-months of follow-up. These results are also confirmed by a randomized controlled trial (RCT) [59] which demonstrated that when the HTs were detached, the SNQ value on MRI increased in the first six months after ACLR and then gradually decreased. In contrast, in the study group where the HT insertion was preserved, the SNQ value remained consistently low, with a significant difference between the two groups at a 12-month follow-up, favoring the study group in which the HT insertion was preserved [59]. Using a methodology similar to that of Vari et al. [56], Cavaignac et al. [60] demonstrated an improvement in MRI indicators of ACL healing when a lateral extra-articular tenodesis (LET) was performed in addition to the ACLR compared with the group where LET was not performed. In both groups, the HTs were detached from their insertion site. The authors hypothesized that reducing stress on the graft when a LET is performed may improve the mechanical environment by reducing overload on the graft and promoting its remodeling. Numerous studies subsequently confirmed the benefits of preserving the insertion of the HTs for graft healing [61,62,63,64,65]. Not only the HTs [55] but also the autologous quadriceps tendon and patellar bone tendon showed a gradual improvement in the parameters at MRI from six months onwards after surgery [66,67] in favor of a more cautious attitude towards proposing an early resumption of loads after ACLR regardless of the graft used. Other modifiable factors have been associated with graft healing, such as lateral tibial slope, remnant preservation, graft orientation and graft/remaining notch volume ratio [68]. Despite previous results reporting an increase of cyclops lesions [69,70], the remnant-sparing ACLR technique is still used today with a minimal rate of complications reported by most recent studies [71,72]. Numerous advantages have been associated with remnant preservation, including greater mechanical stability, better functional results and vascularization, improved remodeling and reduced retear [73,74,75,76,77]. This would be possible due to the presence of mechanoreceptors, vascular stem cells and fibroblasts, which, speculatively, are capable of improving the vascularization, healing and proprioception associated with the graft, in the remnant [71,78]. Furthermore, a recent prospective cohort study displayed how following a correct technique involving the removal of excess tissue from the remnant and then its reduction in volume can allow the remnant-sparing technique to be safely performed without increasing the risk of a cyclops lesion [72]. However, a small remnant size with little graft coverage seems to have no benefit [78]. To maintain extensive remnant leading to increased graft coverage, the optimal timing for conducting this reconstruction is within the initial weeks following ACL injury [74]. However, no differences have been documented in terms of complication with regard to the size of the remnant, suggesting that even a large size can be considered safe [71]. This is confirmed by second-look arthroscopy in which the remnant is able to facilitate graft synovialization when a correct technique is performed [78]. Besides the remnant, other factors have been associated with graft remodeling. An increased lateral tibial plateau slope (LTS) value can negatively influence graft healing, as an increase in LTS increases both shear forces and knee rotation, representing a potential negative factor for the neoligament [68,79]. Graft size is also a topic of debate, as a reduced graft diameter has been associated with early failure, but an excessively large graft diameter relative to the anatomy of the notch has also been linked to impingement within the intercondylar notch, with a risk of stiffness, arthrofibrosis and extension deficit [80,81,82]. Therefore, it is more likely that not the diameter alone but rather the graft/remaining notch volume ratio should be considered to facilitate remodeling and reduce the risk of postoperative complications [69], thus developing a personalized approach [83,84]. An acute graft bending angle (GBA) has also been associated with poorer graft remodeling [82,85], likely due to increased stress at the graft–bone tunnel interface. However, these parameters have a low correlation with clinical outcomes since they are influenced by various factors, including strength, psychological factors, and neuromuscular control [86,87]. Therefore, it is not possible to consider these factors predictive of clinical outcomes.

## 3. Surgery

It is universally accepted that recreating the anatomical tibial and femoral footprint during ACLR is key from a clinical and biomechanical point of view [88,89,90,91,92,93,94,95]. For years, the transtibial (TT) technique has been widely used around the world as the main ACL reconstruction technique. However, the influence of the tibial tunnel on the direction of the femoral tunnel makes it difficult to achieve the correct footprint of the femoral ACL insertion, rendering the TT technique non-anatomical [91,96,97,98]. As such, the anteromedial portal (AMP) and the outside-in (OI) technique have been proposed to better match the native ACL femoral footprint. Compared with the TT technique, the AMP technique demonstrated better reproduction of the anatomical ACL femoral footprint and improved anteroposterior and rotational stability with faster return to activity and better short-term range of motion (ROM) [99,100,101,102,103,104,105,106,107,108,109,110,111,112,113]. However, numerous disadvantages are associated with the AMP technique, many of which are related to demanding techniques, including the need to hyperflex the knee, poor visibility, difficulty in guiding the drill, risk of posterior-wall blowout, cartilage damage to the medial femoral condyle, lesion of the anterior horn of the medial meniscus, damage to the common peroneal nerve in case of distal/inferior beath pin exit and difficulty in visualization in the case of the remnant-sparing technique [99,100,101,102,103,106,109,112,114]. For the outside-in technique, numerous advantages are also described, including the ability to replicate the anatomical ACL footprint, perform the remnant-sparing technique without visibility issues, reduce graft–tunnel mismatch, reduce the risk of posterior-wall blowout and, lastly, facilitate ACL revision [115,116,117,118]. However, some disadvantages have also been reported, such as the need to perform a lateral mini-incision with poorer cosmesis and the risk of injury to lateral knee structures, as well as increased abrasion at the bone–graft interface at the tunnel entrance [102,115,116,117,118]. Several studies have shown similar clinical and functional outcomes, as well as graft healing and failure rates, between the AMP and outside-in techniques [119,120,121,122]. Similarly, no differences in clinical and functional outcomes and graft survival rates were reported for quadriceps tendon (QT), bone–patellar tendon–bone (BPTB) and HT autografts [123,124,125]. However, it has been reported that the ligamentization and remodeling process in HT autografts takes up to one or two years [43], whereas BPTB remodeling ranges between six and twelve months [126].

Beyond the femoral notch, in terms of surgical technique and graft choice, associated injuries must also be considered. In this context, injuries to the meniscal and anterolateral structures increase rotational and anteroposterior instability causing graft overload [127,128,129]. In the case of associated injuries, the reconstruction of the ACL alone does not restore preinjury stability, which causes increased stress on the neoligament. Treatment of associated injuries restores proper rotational stability [128,130]. In fact, combination with a LET has been shown to improve graft healing [60], and reconstruction of the anterolateral ligament (ALL) protects from meniscal repair [24]. As with the ACL, when a meniscal lesion is present, its healing time after repair can no longer be underestimated. Second-look arthroscopy has demonstrated a higher healing rate after 12 months from meniscal repair than at less than 12 months, showing that the healing process of the meniscal injury continues even 1 year after repair, and accelerated physiotherapy protocols have demonstrated higher meniscal repair failure rates than weight-bearing and movement restriction protocols. This indicates that premature stress on the menisci will cause failure of the repair due to insufficient healing to tolerate overloads. In this context, the early overloading of the knee, such as early return to sport, could cause early failure of meniscal repair [131]. A recent study [132] shows that traumatic meniscal injuries can be considered healed one year after repair based on imaging criteria assessed on MRI. However, this must be interpreted with caution, as some injuries such as isolated, complex lesions and bucket handle tears of the meniscus may take longer to heal, being characterized by a high failure rate [133]. Also, most failures occur in the first year due to insufficient healing or new injury [133]. We speculate that the premature overloading of the repaired meniscus may trigger failure of the repair due to the meniscal lesion not yet having fully healed, and it may be advisable to delay overloading and torsional stress by 12 months after ACLR. Due to its protective effect on the menisci, the reconstruction of the anterolateral structures is recommended when performing meniscal repair [134]. A reduction in the failure rate and increased stability are achieved when a modified Lemaire LET is added to the ACLR, due to increased control of rotational stability and a protective effect of the graft [135,136,137,138,139,140,141]. Similarly, combined ACLR and ALL reconstruction, as described by Sonnery-Cottet [142], have been shown to reduce the failure rate to 4.1% while achieving excellent clinical outcomes and better stability and decreasing the need for secondary meniscectomy [134,143].

## 4. Postoperative Recovery

### 4.1. Proprioception

There is contrasting evidence on the recovery of proprioceptive function after ACLR, with most of the papers reporting that full restoration at the baseline is limited and, although there is a progressive recovery over time, maximum proprioceptive performance is often achieved way after the athletes have received clearance for resuming sports activity [144,145].

The clinical relevance of proprioceptive impairment has been extensively investigated, and it has been demonstrated that proprioception and kinesthesia in knee positions close to terminal extension are of paramount importance when the synergic action of passive (ligamentous) and active (contractile) restraints to anterior tibial translation or rotational stress is required [146,147,148].

Altered proprioception leads to changes in arthrokinematics at articular peak loading during dynamic conditions such as jumping, pivoting and landing maneuvers, eventually putting the ACL at risk of failure [149,150]. A recent meta-analysis demonstrated that proprioception is significantly impaired 6 to 24 months after ACLR. In particular, the authors evaluated the proprioceptive function by assessing joint position sense (JPS) and threshold to detection of passive motion (TTDPM) in patients who had undergone ACLR and comparing them to a healthy control group. The ACLR group scored significantly worse than the healthy one [146].

In a case–control study, Suner-Keklick et al. [147], evaluated, in ACLR patients, knee proprioception in the closed kinetic chain position at a targeted angle of 30° degrees of knee flexion. The authors found that in ACLR patients, the proprioceptive sense, both in the ACLR extremity and in the contralateral one, showed inferior outcomes compared with the matched extremity of the control group, even 24 months after surgery [147]. Roberts et al. [148] compared bilateral proprioceptive performance in ACLR patients and healthy age-matched volunteers by using TTDPM from two starting positions (20° and 40°) toward flexion and extension, active reproduction of a 30° passive angle change and visual reproduction of a 30° passive angle change. At a mean of two years from surgery, they observed higher TTDPM both in ACLR and uninjured knees of the study group versus healthy volunteers demonstrating that an impairment in proprioceptive function is present up to two years after ACLR [148].

Moreover, a recent retrospective case–control study compared neuromuscular activation in the lower limb after ACLR with the healthy contralateral one, after a minimum of 3 years from surgery. The main finding was the persistence of a neuromuscular activation deficit of the lower limb (about 42% for the ACLR side compared with the contralateral healthy limb) involving not only the quadriceps muscle but the whole limb musculature [149]. Therefore, novel, holistic approaches are needed and should consider the fact that a knee injury, although localized, can cause global motor consequences. This is supported by McPherson et al. [150], who found that even 12 months after ACLR, patients showed inferior quadriceps and hamstrings motor unit (MU) size and altered firing rate activity in both operated and unoperated limbs compared with healthy controls.

In line with these findings, a longitudinal study evaluated postural regulation and stability over a two-year period in subjects who had undergone ACLR and successive rehabilitation. Postural regulation was measured for stability indicator (ST), weight distribution index (WDI), synchronization (foot coordination) and sway intensities (postural subsystems). The study showed that after ACLR, all the investigated parameters were significantly altered; in fact ACLR determines higher activity of the visual and nigrostriatal systems to compensate for reduced activity of the somatosensory and cerebellar systems. A minimum time period of one year was needed to reach comparable performance to the healthy controls [151].

### 4.2. Functional Recovery

There is evidence in the literature that knee function after ACLR is the last to be recovered [152]. Following ACLR, altered patterns or asymmetries are developed with regard to gait or landing from a jump, which usually represent the most frequent types of movements encountered when playing sport [153,154].

In recent studies, it was reported that at the time of RTP, athletes still presented biomechanical asymmetries (joint angles, moments, and work and muscle forces) during vertical jumps, in the concentric (push-off) phase or during landing from bilateral jumps, despite having passed discharge criteria [155,156]. It has been reported that having multiplanar biomechanical asymmetries of the hip and the knee bears a three times higher risk for second ACL injury within 1 year from ACLR compared with those subjects who do not display such asymmetries [157].

In a recent study, Sharafoddin-Shiraz et al. [158] examined longitudinally for 2 years, with 6-month intervals, asymmetries in double-leg landing kinetics and kinematics of subjects who had undergone ACLR for unilateral ACL rupture, comparing these patients with healthy controls. The authors reported that patients showed altered kinematic and kinetic parameters (higher peak hip and lower knee and ankle flexion moments and higher peak knee adduction, as well as higher hip flexion angles and moments and hip abduction angles on the surgical side, compared with the uninjured side and controls) at all follow-ups prior to 24 months, at which point the asymmetries were no longer present [157].

In another recent study, single-limb landing biomechanics were evaluated over time (10 months and 3 years) in a patient group who had undergone ACLR [159]. Results from the study showed asymmetries in landing that persisted over a 3-year observation period (reduced knee flexion angles and moments on the ACLR knee and increased hip flexion moment compared with the contralateral side). It should be noted that reduced knee flexion is a pretty consistent finding of studies investigating single-limb landing biomechanics after ACLR [160]. This data takes on particular importance considering that the more extended the knee is during landing the more it potentially loads the ACL through a quadriceps-driven anterior tibial shear forces, eventually leading to a higher risk of reinjury [161].

To compensate for reduced knee flection, patients increase hip flexion moment. This strategy presents since the early postoperative time and persists for a long period. The clinical implications for the overloaded hip joint are currently unknown [162]. A recent cross-sectional study evaluated motor control recovery, comparing asymptomatic athletes with an ACLR group at different time points after surgery (early: <6 months; mid: 6–18 months; late: ≥18 months). In particular, in a stair descent task, the “temporal symmetry” during the stance subphases (single-support, first and second double-support) and the “spatial symmetry” for hip–knee–ankle intra-joint angular displacements during the stance phase were evaluated. There were significant differences between groups in temporal measures showing asymmetry at the early- and mid-follow-ups (up to 18 months following surgery) compared with the late one, specifically during the first double-support-phase durations (longer for their injured vs. non-injured leg) [163]. The study demonstrated that temporal but not spatial asymmetries often arise early after ACLR and persist for up to 18 months. These alterations could be present in the late rehabilitation stages or even beyond and may contribute to increased risk of reinjury in the ACLR population [164].

In addition, gait analysis studies have shown that functional asymmetries in knee joint kinematics during walking can persist in the long term—two or more years after ACL reconstruction—with decreased movement amplitudes in the operated knee still evident even eight years or more post-surgery [33,165].

### 4.3. Strength

Recovering muscle strength of the thigh in individuals after ACLR is a crucial aspect of rehabilitation because of their primary role in providing dynamic knee stability and in increasing the likelihood of returning to knee-demanding activities. A recent meta-analysis evaluated the isometric muscle strength and activation, measured by central activation ratios (CARs), of the quadriceps muscle between the operated limb and contralateral limb of patients who had undergone ACLR and then compared these outcomes with healthy controls. The authors found that the operated limb of the ACLR subjects showed lower knee extension strength when compared with the unoperated limb and the healthy controls. The CAR values were similar between limbs in the ACLR patients but lower when comparing both limbs of ACLR patients versus healthy volunteers. This means that strength deficit of the operated limb and muscle activation deficits of both limbs persist for years after ACLR [166].

Central and peripheral modifications have been proposed as contributors to persistent alterations in quadriceps function after ACLR. This process, globally known as arthrogenic muscle inhibition (AMI), causes neural-based inhibition of muscle activation originating from a series of modifications involving impairment in muscle resting motor thresholds, altered articular sensory receptors and altered spinal reflex excitability, together with intracortical inhibition [167]. These deficits, persisting for years after surgery, may contribute to altered movement patterns, leading to a greater risk of developing a second ACL injury. Specific interventions, such as transcutaneous electrical nerve stimulation in conjunction with therapeutic exercise, acting through the disinhibition of neural mechanisms, have been shown to help improve strength in individuals after ACLR [168].

The literature appears equivocal on the effects of graft type on the recovery of quadriceps strength after ACLR. A meta-analysis of level III evidence studies investigated the influence of graft choice on isokinetic muscle strength 4 to 24 months following ACLR [169]. The study showed that isokinetic muscle strength is influenced by the donor site for ACLR; in addition, these deficits persist up to two years after ACLR. In particular, athletes who underwent ACLR with a BPTB autograft demonstrated a greater knee extensor deficit and lower knee flexor deficits than patients with an HT autograft [169]. Conversely, Aglietti et al. [170], in an RCT, measured concentric knee extensor and flexor muscles at different angular velocities with an isokinetic dynamometer in a sample of 120 athletes to compare BPTB vs. HT autografts at 4, 12, and 24 months. At the 2-year follow-up evaluation, the knee extensor and flexor strength of the operated limb was comparable to that of the contralateral side. Furthermore, the authors reported a marked improvement in extensor strength regardless of the graft type [170]. Inagaki et al. showed comparable symmetry to that of an uninjured population in average peak isokinetic knee extensor and flexor measurements in patients two years after ACLR with isolated semitendinosus or combined semitendinosus and gracilis tendons [171]. Conversely Chatzilamprinos et al. [172] in a recent study, demonstrated that two years after ACLR, soccer players continue to show deficits in both strength and functional performance (hop test). The isokinetic evaluation displayed significant deficits of the knee extensors’ concentric peak torque for the operated limb compared with the healthy one, and a large percentage of participants did not achieve the required symmetry (90–110%) for the knee extensors and flexors between the two limbs.

A previous meta-analysis compared quadriceps strength between the injured limb of individuals with ACLR and the limb of age-, sex- and activity-matched healthy controls. The results showed that regardless of time from surgery (within 6 months, 6 to 18 months and >18 months up to 4 years after ACLR), individuals with ACLR had lower quadriceps strength in their injured limb compared with the matched healthy controls. The outcomes from the study clearly show that quadriceps strength impairment persists over several years after ACLR compared with appropriately matched healthy controls. Therefore, the current return-to-sports criteria that rely basically on the LSI may need to be reconsidered, and comparing ACLR patients to a matched control group might be more appropriate [173]. In a cross-sectional comparison, Ageberg et al. [174] evaluated the quadriceps and hamstring muscle power in patients who had undergone ACLR reconstruction with a BPTB or HT graft at a mean of three years after surgery. The main result of this study was the lower hamstring muscle power, and the lower hamstring-to-quadriceps ratio in the HT graft group than in the BPTB graft group three years (range two to five years) after ACLR. Such a finding reflects the imbalance of knee muscles within the injured leg and between legs after reconstruction with an HT graft. Thus, the ability of the hamstring muscles to protect the knee after ACLR with an HT graft may be impaired, with a possible negative effect on dynamic knee joint stabilization and a possible higher risk of graft rupture.

In another recent meta-analysis, Hogberg J. et al. [175] assessed the recovery of knee flexor strength in patients who had undergone ACLR with an HT graft. The main finding was the incomplete recovery of knee flexor strength symmetry in the first year after ACLR, with regards to the reference cut-off value of 90% LSI. Furthermore, it took up to two years to achieve the LSI in isokinetic knee flexor strength after ACL reconstruction at both 60°/s and 180°/s of angular velocity.

## 5. Conclusions

It has been reported that most of the cases of reinjury occur within two years from ACLR. This data can be explained by numerous pieces of evidence showing that the recovery of proprioception, proper neuromuscular activation and strength, as well as proper biomechanics, remains affected for a long time after surgery (up to two or three years in some cases) despite adequate rehabilitation programs. According to the current literature, a period of two years should be considered before resuming high-impact sports activities. This can be challenging for a young athlete to accept because of the impact on their career. However, the athlete who returns to sport within the common time frame of 6 to 12 months is exposed to a high risk of reinjury. The growing body of evidence supporting a more cautious RTS often contrasts with the strong pressures for early return to play, particularly among professional athletes.

Educating both the athlete and their support team about the risks associated with premature RTS currently appears to be the most effective strategy. A deeper understanding of the healing dynamics is required—first and foremost, gaining more detailed insights into the biological timelines of healing and maturation of the reconstructed structures (i.e., the ACL graft), as well as the repaired structures (such as commonly associated meniscal tears). Moreover, optimizing rehabilitation strategies through the use of emerging technologies may allow for the more precise identification of biomechanical (kinetic and kinematic) deficits, which represent weak links and recognized risk factors for graft failure and reinjury.

## Data Availability

The datasets used and/or analyzed during the current study are included in bibliography of this published article.

## Abbreviations

ACL	anterior cruciate ligament
ACLR	anterior cruciate ligament reconstruction
RTP	return to play
HT	hamstring tendon
MRI	magnetic resonance imaging
SNQ	signal-to-noise quotient
RCT	randomized controlled trial
LET	lateral extra-articular tenodesis
LTS	lateral tibial plateau slope
GBA	graft bending angle
AMP	anteromedial portal
OI	outside-in
TT	transtibial
ROM	range of motion
QT	quadriceps tendon
BPTB	bone–patellar tendon–bone
ALL	anterolateral ligament
TTDPM	threshold to detection of passive motion
MU	motor unit
ST	stability indicator
WDI	weight distribution index
AMI	arthrogenic muscle inhibition
CAR	central activation ratio
LSI	limb symmetry index
